# Note on Coarse Alignment of Gyro-Free Inertial Navigation System

**DOI:** 10.3390/s23125763

**Published:** 2023-06-20

**Authors:** Heyone Kim, Jae-Hoon Son, Sang-Heon Oh, Dong-Hwan Hwang

**Affiliations:** 1 Advanced Technology Research Team, Hyundai Rotem, Uiwang-si 16082, Republic of Korea; heyone.kim@hyundai-rotem.co.kr; 2 Department of Electronics Engineering, Chungnam National University, Daejeon 34134, Republic of Korea; sonjaehoon@cnu.ac.kr; 3 Advanced Technology Laboratory, Microinfinity Co., Ltd., Daejeon 34012, Republic of Korea; laborosh@minfinity.com

**Keywords:** coarse alignment, gyro-free inertial navigation system, distributed accelerometers, heading

## Abstract

In this note, the feasibility of initial alignment of a gyro-free inertial navigation system (GF-INS) is investigated. Initial roll and initial pitch are obtained using leveling of conventional INS since centripetal acceleration is very small. The equation for the initial heading cannot be used since the GF inertial measurement unit (IMU) cannot directly measure the Earth rate. A new equation is derived to obtain the initial heading from GF-IMU accelerometer outputs. Initial heading is expressed in the accelerometer outputs of two configurations, which satisfies a specific condition among 15 GF-IMU configurations presented in the literature. The initial heading error to arrangement and accelerometer error is quantitatively analyzed from the initial heading calculation equation of GF-INS and the initial heading error analysis of the general INS. The initial heading error is investigated when gyroscopes are used with GF-IMU. The results show that the initial heading error depends more on the performance of the gyroscope than that of the accelerometer, and the initial heading cannot be obtained within a practical error level by using only GF-IMU, even when an extremely accurate accelerometer is used. Therefore, aiding sensors have to be used in order to have a practical initial heading.

## 1. Introduction

The inertial navigation system (INS) continuously provides the attitude, velocity, and position of a vehicle from the specific force and angular velocity measured by an inertial measurement unit (IMU) [1,2,3]. Since the INS has a high output rate and wide bandwidth, it has been widely used as a navigation system of vehicles with high dynamics, such as rockets, guided missiles and aircraft [4].

The initial attitude, initial velocity, and initial position have to be known before the navigation is carried out since the INS calculates a navigation solution by integrating IMU measurements. The initial velocity and initial position have be externally provided, while the initial attitude can be determined in stationary by itself from the gravity and the Earth rate measured by the IMU [2]. This initial attitude determination without external aiding is called self-alignment [5]. In the gimbaled INS (GINS), the self-alignment is a procedure that aligns physically the platform with the navigation frame using gimbals. The self- alignment in the strap-down INS (SDINS) is a procedure to determine the initial attitude represented by the direction cosine matrix (DCM) between the body frame and the navigation frame or Euler angles, roll, pitch, and heading.

The GF-INS is an inertial navigation system that uses only accelerometers. Shuler first proposed in the 1960s the idea that measures the angular motion of a vehicle using the lever-arm effect of the accelerometer [6]. However, subsequent research was not followed since the quality of the accelerometer was not good enough to completely replace the gyroscope. With the development of micro-electro-mechanical system (MEMS) technology, nanotechnology, and cold atomic technology from the late 1990s, researchers have paid much attention to the GF-INS [7,8].

Before performing navigation, attitude should be initialized in the GF-INS as the conventional INS. Even though the self-alignment algorithm for the conventional INS is available, it cannot be used without modification since the measuring method of angular motion for a vehicle in the GF-INS is fundamentally different from that of the conventional INS.

Vaknin and Klein proposed a partial coarse alignment procedure for the roll and pitch only and developed closed-form expressions of the attitude error of the GF-INS coarse alignment [9]. They compared the partial coarse alignment performances of several GF configurations. Vaknin and Klein claimed that a useful initial heading cannot be obtained, regardless of the accelerometer grade, since the GF-INS cannot directly measure the Earth rate [9].

In this note, a coarse alignment of the GF-INS is presented, and the feasibility of the initial heading is evaluated. The initial heading expression in accelerometer outputs of the GF-IMU is derived from the initial heading expression in the coarse alignment of the conventional INS. The configurations of the GF-IMU, in which the initial heading expression can be obtained, are investigated for the configurations of the GF-IMU in the literature [9,10,11,12,13,14,15,16,17]. The initial heading error to arrangement and accelerometer error is quantitatively analyzed from the initial heading calculation equation of GF-INS and the initial heading error analysis of the general INS. The initial heading error is investigated when gyroscopes are used with GF-IMU.

The organization of this note is as follows: in Section 2, the coarse alignment for the conventional INS is introduced. In Section 3, the coarse alignment of the GF-INS is described. In Section 4, the coarse alignment of the GF-INS is described when gyroscopes are added to the GF-IMU. Finally, concluding remarks and further studies are presented in Section 5.

## 2. Coarse Alignment of INS

### 2.1. Leveling

Only gravity is measured by the accelerometer when the vehicle is stationary. The measured gravity is represented in Equation (1) between the body frame and the navigation frame [18,19,20].

(1) $$\begin{array}{cc} f^{b} & =C_{n}^{b}f^{n}=C_{3}C_{2}C_{1}f^{n}\\ & =\left[\begin{array}{ccc} 1 & 0 & 0\\ 0 & \cos\phi & \sin\phi \\ 0 & -\sin\phi & \cos\phi \end{array}\right]\left[\begin{array}{ccc} \cos\theta & 0 & -\sin\theta \\ 0 & 1 & 0\\ \sin\theta & 0 & \cos\theta \end{array}\right]\left[\begin{array}{ccc} \cos\psi & \sin\psi & 0\\ -\sin\psi & \cos\psi & 0\\ 0 & 0 & 1 \end{array}\right]\left[\begin{array}{c} 0\\ 0\\ -g \end{array}\right]\\ & =\left[\begin{array}{ccc} \cos\theta \cos\psi & \cos\theta \sin\psi & -\sin\theta \\ \sin\phi \sin\theta \cos\psi -\cos\phi \sin\psi & \sin\phi \sin\theta \sin\psi +\cos\phi \cos\psi & \sin\phi \cos\theta \\ \cos\phi \sin\theta \cos\psi +\sin\phi \sin\psi & \cos\phi \sin\theta \sin\psi -\sin\phi \cos\psi & \cos\phi \cos\theta \end{array}\right]\left[\begin{array}{c} 0\\ 0\\ -g \end{array}\right]\\ & =\left[\begin{array}{c} g\sin\theta \\ -g\sin\phi \cos\theta \\ -g\cos\phi \cos\theta \end{array}\right]=\left[\begin{array}{c} {\left(f^{b}\right)}_{x}\\ {\left(f^{b}\right)}_{y}\\ {\left(f^{b}\right)}_{z} \end{array}\right] \end{array}$$

where ϕ, θ, and ψ represent roll, pitch, and heading, respectively. The symbol g denotes gravity. The specific force is represented in the body frame and the navigation frame as $f^{b}$ and $f^{n}$, respectively. $C_{n}^{b}$ denotes the DCM between the navigation frame and the body frame. $C_{1}$, $C_{2},$ and $C_{3}$ correspond to DCMs between two frames when the frame is rotated ψ, θ, and ϕ around the yaw, pitch, and roll axes, respectively.

Roll can be obtained as Equation (2) from Equation (1) [18,19,20].

(2) $$\phi =\tan^{-1}\left(\frac{{\left(f^{b}\right)}_{y}}{{\left(f^{b}\right)}_{z}}\right)$$

where ${\left(f^{b}\right)}_{y}$ and ${\left(f^{b}\right)}_{z}$ are the y-axis and the z-axis component of the specific force, respectively. Pitch θ is obtained from Equation (3) [18,19,20].

(3) $$\theta =\tan^{-1}\left(\frac{{\left(f^{b}\right)}_{x}}{\sqrt{{\left(f^{b}\right)}_{y}^{2}+{\left(f^{b}\right)}_{z}^{2}}}\right)$$

where ${\left(f^{b}\right)}_{x}$ is the x-axis component of the specific force.

### 2.2. Gyrocompassing

Only the Earth rate is measured by the gyroscope when the vehicle is stationary. The Earth rate is represented as Equation (4) in the body frame and in the navigation frame [18,19,20].

(4) $$\omega_{ie}^{b}=C_{n}^{b}\omega_{ie}^{n}=C_{3}C_{2}C_{1}\left[\begin{array}{c} \Omega \cos L\\ 0\\ -\Omega \sin L \end{array}\right]$$

where Ω and L are the magnitude of the Earth rate and the latitude, respectively. $\omega_{ie}^{b}$ and $\omega_{ie}^{n}$ are the Earth rate represented in the body frame and navigation frame, respectively. From Equation (4), the Earth rate $\omega_{ie}^{r}$ represented in the r frame, which is rotated from the navigation frame by the heading ψ, is given in Equation (5) [18,19,20].

(5) $$\begin{array}{cc} \omega_{ie}^{r} & =C_{1}\omega_{ie}^{n}=\left[\begin{array}{c} \Omega \cos L\cos\psi \\ -\Omega \cos L\sin\psi \\ -\Omega \sin L \end{array}\right]\\ & ={\left(C_{3}C_{2}\right)}^{-1}\omega_{ie}^{b}=\left[\begin{array}{c} {\left(\omega_{ie}^{b}\right)}_{x}\cos\theta +{\left(\omega_{ie}^{b}\right)}_{y}\sin\phi \sin\theta +{\left(\omega_{ie}^{b}\right)}_{z}\cos\phi \sin\theta \\ {\left(\omega_{ie}^{b}\right)}_{y}\cos\phi -{\left(\omega_{ie}^{b}\right)}_{z}\sin\phi \\ -{\left(\omega_{ie}^{b}\right)}_{x}\sin\theta +{\left(\omega_{ie}^{b}\right)}_{y}\sin\phi \cos\theta +{\left(\omega_{ie}^{b}\right)}_{z}\cos\phi \cos\theta \end{array}\right]=\left[\begin{array}{c} {\left(\omega_{ie}^{r}\right)}_{x}\\ {\left(\omega_{ie}^{r}\right)}_{y}\\ {\left(\omega_{ie}^{r}\right)}_{z} \end{array}\right] \end{array}$$

From Equation (5), the heading is obtained from Equation (6) [18,19,20].

(6) $$\psi =\tan^{-1}\left(-\frac{{\left(\omega_{ie}^{r}\right)}_{y}}{{\left(\omega_{ie}^{r}\right)}_{x}}\right)=\tan^{-1}\left(-\frac{{\left(\omega_{ie}^{b}\right)}_{y}\cos\phi -{\left(\omega_{ie}^{b}\right)}_{z}\sin\phi }{{\left(\omega_{ie}^{b}\right)}_{x}\cos\theta +{\left(\omega_{ie}^{b}\right)}_{y}\sin\phi \sin\theta +{\left(\omega_{ie}^{b}\right)}_{z}\cos\phi \sin\theta }\right)$$

## 3. Coarse Alignment of GF-INS

### 3.1. Roll and Pitch

The k-th accelerometer output of the GF-IMU with N accelerometers is given in Equation (7) [11].

(7) $$y_{k}={\left[d_{k}^{b}\right]}^{T}f_{k}^{b}={\left[d_{k}^{b}\right]}^{T}\left[a^{b}+\omega_{ib}^{b}\times \left(\omega_{ib}^{b}\times r_{k}^{b}\right)+{\dot{\omega }}_{ib}^{b}\times r_{k}^{b}-g^{b}\right]$$

where $d_{k}^{b}$ denotes the sensing direction of the k-th accelerometer. $f_{k}^{b}$ is the specific force of the k-th accelerometer represented in the body frame. $a^{b}$ is the acceleration at the center of the gravity of the vehicle represented in the body frame. $\omega_{ib}^{b}$ is the angular velocity of the body frame with respect to the inertial frame represented in the body frame. ${\dot{\omega }}_{ib}^{b}$ is the angular acceleration of the body frame with respect to the inertial frame represented in the body frame. $r_{k}^{b}$ is the position of the k-th accelerometer represented in the body frame. $g^{b}$ is the gravity vector represented in the body frame. If the vehicle is stationary on Earth, then $a^{b}=0$, ${\dot{\omega }}_{ib}^{b}=0,$ and $\omega_{ib}^{b}=\omega_{ie}^{b}+\omega_{eb}^{b}=\omega_{ie}^{b}$. Therefore, the k-th accelerometer output can be represented in Equation (8).

(8) $$y_{k}={\left[d_{k}^{b}\right]}^{T}f_{k}^{b}={\left[d_{k}^{b}\right]}^{T}\left[\omega_{ie}^{b}\times \left(\omega_{ie}^{b}\times r_{k}^{b}\right)-g^{b}\right]$$

In Equation (8), the magnitude of the centripetal acceleration is much less than the gravity as Equation (9) since the magnitude of the vector $\omega_{ie}^{b}$, $\left\|\omega_{ie}^{b}\right\|,$ is $7.292115\times 10^{-5}\;\mathrm{rad}/$s [9].

(9) $$\left\|\omega_{ie}^{b}\times (\omega_{ie}^{b}\times r_{k}^{b})\right\|\ll \left\|g^{b}\right\|$$

As a result of this, Equation (8) can be represented in Equation (10) [9].

(10) $$y_{k}\approx [d_{k}^{b}{]}^{T}f_{k}^{b}=[d_{k}^{b}{]}^{T}\left(-g^{b}\right)$$

The calculated specific force ${\hat{f}}^{b}$ from N accelerometers outputs can be obtained from Equation (11).

(11) $${\hat{f}}^{b}={\left[\begin{array}{ccc} {\left({\hat{f}}^{b}\right)}_{x} & {\left({\hat{f}}^{b}\right)}_{y} & {\left({\hat{f}}^{b}\right)}_{z} \end{array}\right]}^{T}=-{\hat{g}}^{b}={\left(D^{T}D\right)}^{-1}D^{T}\tilde{y}$$

where D is the sensing direction matrix of the accelerometers in Equation (12).

(12) $$D={\left[\begin{array}{cccc} {\left(d_{1}^{b}\right)}^{T} & {\left(d_{2}^{b}\right)}^{T} & \cdot \cdot \cdot & {\left(d_{N}^{b}\right)}^{T} \end{array}\right]}^{T}$$

$\tilde{y}$ is the accelerometer measurement vector in Equation (13).

(13) $$\tilde{y}={\left[\begin{array}{cccc} {\tilde{y}}_{1} & {\tilde{y}}_{2} & \cdots & {\tilde{y}}_{N} \end{array}\right]}^{T}$$

Inserting the specific force in Equation (11) into Equations (2) and (3), roll and pitch can be obtained.

### 3.2. Heading

In order to obtain the heading, let us change Equation (6) to Equation (14).

(14) $$\begin{array}{cc} \psi & =\tan^{-1}\left(-\frac{{\left(\omega_{ie}^{r}\right)}_{y}}{{\left(\omega_{ie}^{r}\right)}_{x}}\right)=\tan^{-1}\left(-\frac{{\left(\omega_{ie}^{b}\right)}_{x}{\left(\omega_{ie}^{r}\right)}_{y}}{{\left(\omega_{ie}^{b}\right)}_{x}{\left(\omega_{ie}^{r}\right)}_{x}}\right)\\ & =\tan^{-1}\left(-\frac{{\left(\omega_{ie}^{b}\right)}_{x}{\left(\omega_{ie}^{b}\right)}_{y}\cos\phi -{\left(\omega_{ie}^{b}\right)}_{x}{\left(\omega_{ie}^{b}\right)}_{z}\sin\phi }{{\left(\omega_{ie}^{b}\right)}_{x}^{2}\cos\theta +{\left(\omega_{ie}^{b}\right)}_{x}{\left(\omega_{ie}^{b}\right)}_{y}\sin\phi \sin\theta +{\left(\omega_{ie}^{b}\right)}_{x}{\left(\omega_{ie}^{b}\right)}_{z}\cos\phi \sin\theta }\right) \end{array}$$

Equation (8) can be expressed as Equation (15).

(15) $$\begin{array}{cc} y_{k}+[d_{k}^{b}{]}^{T}g^{b} & =[d_{k}^{b}{]}^{T}f_{k}^{b}+[d_{k}^{b}{]}^{T}g^{b}\\ & =[d_{k}^{b}{]}^{T}[\omega_{ie}^{b}\times (\omega_{ie}^{b}\times r_{k}^{b})]=[d_{k}^{b}{]}^{T}\Omega_{ie}^{b}\Omega_{ie}^{b}r_{k}^{b}\\ & ={\left[\begin{array}{c} {\left(d_{k}^{b}\right)}_{x}\\ {\left(d_{k}^{b}\right)}_{y}\\ {\left(d_{k}^{b}\right)}_{z} \end{array}\right]}^{T}\left[\begin{array}{ccc} 0 & -{\left(\omega_{ie}^{b}\right)}_{z} & {\left(\omega_{ie}^{b}\right)}_{y}\\ {\left(\omega_{ie}^{b}\right)}_{z} & 0 & -{\left(\omega_{ie}^{b}\right)}_{x}\\ -{\left(\omega_{ie}^{b}\right)}_{y} & {\left(\omega_{ie}^{b}\right)}_{x} & 0 \end{array}\right]\left[\begin{array}{ccc} 0 & -{\left(\omega_{ie}^{b}\right)}_{z} & {\left(\omega_{ie}^{b}\right)}_{y}\\ {\left(\omega_{ie}^{b}\right)}_{z} & 0 & -{\left(\omega_{ie}^{b}\right)}_{x}\\ -{\left(\omega_{ie}^{b}\right)}_{y} & {\left(\omega_{ie}^{b}\right)}_{x} & 0 \end{array}\right]\left[\begin{array}{c} (r_{k}^{b}{)}_{x}\\ (r_{k}^{b}{)}_{y}\\ (r_{k}^{b}{)}_{z} \end{array}\right]\\ & ={\left[\begin{array}{c} {\left(d_{k}^{b}\right)}_{x}\\ {\left(d_{k}^{b}\right)}_{y}\\ {\left(d_{k}^{b}\right)}_{z} \end{array}\right]}^{T}\left[\begin{array}{ccc} -{\left(\omega_{ie}^{b}\right)}_{y}^{2}-{\left(\omega_{ie}^{b}\right)}_{z}^{2} & {\left(\omega_{ie}^{b}\right)}_{x}{\left(\omega_{ie}^{b}\right)}_{y} & {\left(\omega_{ie}^{b}\right)}_{x}{\left(\omega_{ie}^{b}\right)}_{z}\\ {\left(\omega_{ie}^{b}\right)}_{x}{\left(\omega_{ie}^{b}\right)}_{y} & -{\left(\omega_{ie}^{b}\right)}_{x}^{2}-{\left(\omega_{ie}^{b}\right)}_{z}^{2} & {\left(\omega_{ie}^{b}\right)}_{y}{\left(\omega_{ie}^{b}\right)}_{z}\\ {\left(\omega_{ie}^{b}\right)}_{x}{\left(\omega_{ie}^{b}\right)}_{z} & {\left(\omega_{ie}^{b}\right)}_{y}{\left(\omega_{ie}^{b}\right)}_{z} & -{\left(\omega_{ie}^{b}\right)}_{x}^{2}-{\left(\omega_{ie}^{b}\right)}_{y}^{2} \end{array}\right]\left[\begin{array}{c} (r_{k}^{b}{)}_{x}\\ (r_{k}^{b}{)}_{y}\\ (r_{k}^{b}{)}_{z} \end{array}\right] \end{array}$$

where ${\left(\omega_{ie}^{b}\right)}_{x}$, ${\left(\omega_{ie}^{b}\right)}_{y}$, and ${\left(\omega_{ie}^{b}\right)}_{z}$ are the x-, y−, and z-axis component of $\omega_{ie}^{b}$, respectively. $\Omega_{ie}^{b}$ is the skew symmetric matrix of the vector $\omega_{ie}^{b}$. ${\left(d_{k}^{b}\right)}_{x}$, ${\left(d_{k}^{b}\right)}_{y}$, and ${\left(d_{k}^{b}\right)}_{z}$ are the x−, y−, and z-axis component of the sensing direction of the k-th accelerometer, respectively.

Rearranging Equation (15), Equation (16) can be obtained.

(16) $$\begin{array}{c} y_{k}+{\left[d_{k}^{b}\right]}^{T}g^{b}={\left[\begin{array}{c} (r_{k}^{b}{)}_{y}{\left(d_{k}^{b}\right)}_{x}+(r_{k}^{b}{)}_{x}{\left(d_{k}^{b}\right)}_{y}\\ (r_{k}^{b}{)}_{z}{\left(d_{k}^{b}\right)}_{x}+(r_{k}^{b}{)}_{x}{\left(d_{k}^{b}\right)}_{z}\\ (r_{k}^{b}{)}_{z}{\left(d_{k}^{b}\right)}_{y}+(r_{k}^{b}{)}_{y}{\left(d_{k}^{b}\right)}_{z}\\ -(r_{k}^{b}{)}_{y}{\left(d_{k}^{b}\right)}_{y}-(r_{k}^{b}{)}_{z}{\left(d_{k}^{b}\right)}_{z}\\ -(r_{k}^{b}{)}_{x}{\left(d_{k}^{b}\right)}_{x}-(r_{k}^{b}{)}_{z}{\left(d_{k}^{b}\right)}_{z}\\ -(r_{k}^{b}{)}_{x}{\left(d_{k}^{b}\right)}_{x}-(r_{k}^{b}{)}_{y}{\left(d_{k}^{b}\right)}_{y} \end{array}\right]}^{T}\left[\begin{array}{c} {\left(\omega_{ie}^{b}\right)}_{x}{\left(\omega_{ie}^{b}\right)}_{y}\\ {\left(\omega_{ie}^{b}\right)}_{x}{\left(\omega_{ie}^{b}\right)}_{z}\\ {\left(\omega_{ie}^{b}\right)}_{y}{\left(\omega_{ie}^{b}\right)}_{z}\\ {\left(\omega_{ie}^{b}\right)}_{x}^{2}\\ {\left(\omega_{ie}^{b}\right)}_{y}^{2}\\ {\left(\omega_{ie}^{b}\right)}_{z}^{2} \end{array}\right]\\ \;\;\;\;\;\;\;\;\;\;\;\;\;\;\;\;\;\;=\left[\begin{array}{cccccc} {\left(h_{k}\right)}_{1} & {\left(h_{k}\right)}_{2} & {\left(h_{k}\right)}_{3} & {\left(h_{k}\right)}_{4} & {\left(h_{k}\right)}_{5} & {\left(h_{k}\right)}_{6} \end{array}\right]x \end{array}$$

where x is given in Equation (17).

(17) $$\begin{array}{cc} x & ={\left[\begin{array}{cccccc} x_{1} & x_{2} & x_{3} & x_{4} & x_{5} & x_{6} \end{array}\right]}^{T}\\ & ={\left[\begin{array}{cccccc} {\left(\omega_{ie}^{b}\right)}_{x}{\left(\omega_{ie}^{b}\right)}_{y} & {\left(\omega_{ie}^{b}\right)}_{x}{\left(\omega_{ie}^{b}\right)}_{z} & {\left(\omega_{ie}^{b}\right)}_{y}{\left(\omega_{ie}^{b}\right)}_{z} & {\left(\omega_{ie}^{b}\right)}_{x}^{2} & {\left(\omega_{ie}^{b}\right)}_{y}^{2} & {\left(\omega_{ie}^{b}\right)}_{z}^{2} \end{array}\right]}^{T} \end{array}$$

If all the N accelerometer outputs of the GF-MU are put together, Equation (18) can be obtained from Equation (16).

(18) $$\begin{array}{cc} a & =Hx={\left[\begin{array}{cccc} a_{1} & a_{2} & \cdots & a_{N} \end{array}\right]}^{T}\\ & =\left[\begin{array}{c} y_{1}+{\left[d_{1}^{b}\right]}^{T}g^{b}\\ \vdots \\ y_{N}+{\left[d_{N}^{b}\right]}^{T}g^{b} \end{array}\right]=\left[\begin{array}{cccccc} {\left(h_{1}\right)}_{1} & {\left(h_{1}\right)}_{2} & {\left(h_{1}\right)}_{3} & {\left(h_{1}\right)}_{4} & {\left(h_{1}\right)}_{5} & {\left(h_{1}\right)}_{6}\\ \vdots & \vdots & \vdots & \vdots & \vdots & \vdots \\ {\left(h_{N}\right)}_{1} & {\left(h_{N}\right)}_{2} & {\left(h_{N}\right)}_{3} & {\left(h_{N}\right)}_{4} & {\left(h_{N}\right)}_{5} & {\left(h_{N}\right)}_{6} \end{array}\right]x \end{array}$$

From Equation (18), x can be obtained as Equation (19).

(19) $$x={\left(H^{T}H\right)}^{-1}H^{T}a$$

The heading can be expressed as Equation (20) from Equation (14) and x of Equation (19).

(20) $$\psi =\tan^{-1}\left(-\frac{x_{1}\cos\phi -x_{2}\sin\phi }{x_{4}\cos\theta +x_{1}\sin\phi \sin\theta +x_{2}\cos\phi \sin\theta }\right)$$

### 3.3. Accelerometer Configuration and Possiblity of Initial Heading Calculation

In this section, it is investigated, using Equation (20) in Section 3.2, whether the initial heading can be calculated for the accelerometer arrangements of GF-IMUs. It is known that at least six accelerometers are required to obtain the navigation information of a rigid body motion using accelerometers [10]. The navigation performance of a GF-INS is known to depend on the arrangement of the accelerometers, including the number, location, and sensing direction of the accelerometers. These results are found in references [9,10,11,12,13,14,15,16,17]. In this note, the possibility of the coarse alignment is investigated for the arrangements with 6 to 12 accelerometers, which have been considered in the existing research results.

**Figure 1:**
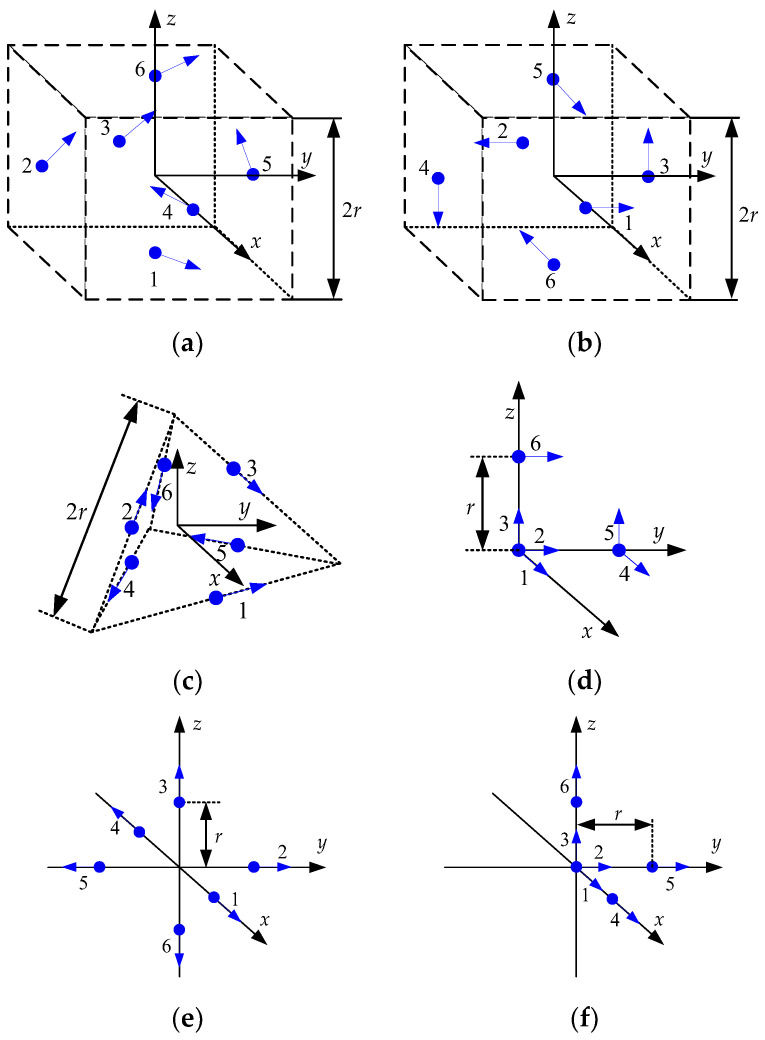
Six accelerometers arrangements for GF-INS. (**a**) Configuration 1, (**b**) Configuration 2, (**c**) Configuration 3, (**d**) Configuration 4, (**e**) Configuration 5, (**f**) Configuration 6.

**Figure 2:**
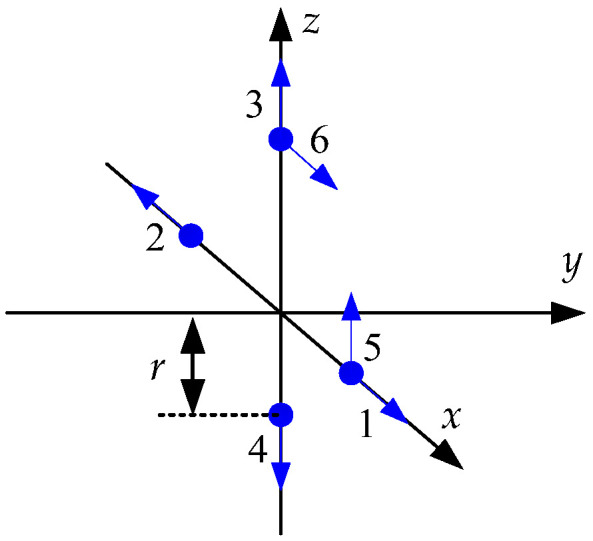
Seven accelerometers arrangement for GF-INS. Configuration 7.

**Figure 3:**
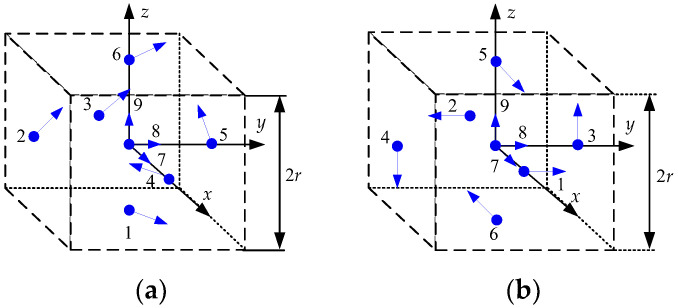

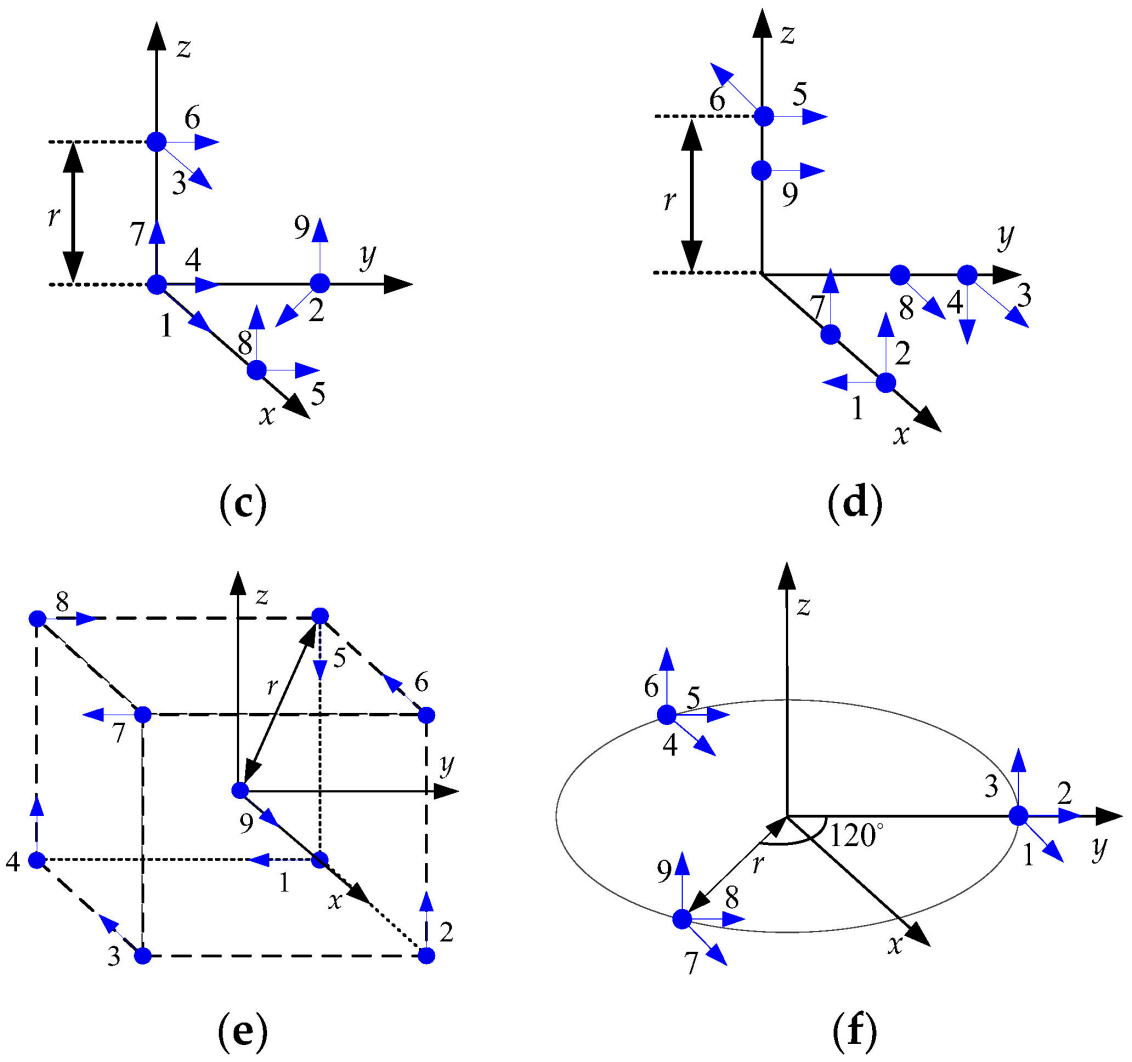
Nine accelerometers arrangements for GF-INS. (**a**) Configuration 8, (**b**) Configuration 9, (**c**) Configuration 10, (**d**) Configuration 11, (**e**) Configuration 12, (**f**) Configuration 13.

**Figure 4:**
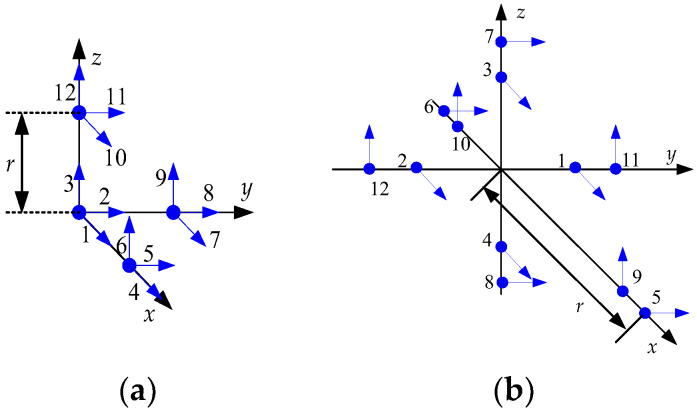
Twelve accelerometers arrangements for GF-INS. (**a**) Configuration 14, (**b**) Configuration 15.

Figure 1, Figure 2, Figure 3 and Figure 4 show 6 arrangements with 6 accelerometers, 1 arrangement with 7 accelerometers, 6 arrangements with 9 accelerometers, and 2 arrangements with 12 accelerometers of GF-IMUs, respectively. In Figure 1, Figure 2, Figure 3 and Figure 4, the black arrows indicate the x−, y−, and z-axes of the body frame. Blue dots indicate the positions of accelerometers and blue arrows the sensing directions of accelerometers.

If x can be obtained from Equation (18), the initial heading can be obtained from Equation (19). In order to obtain x, the rank of the matrix $H^{T}H$ has to be 6. The ranks of the matrix $H^{T}H$ for the arrangements in Figure 1, Figure 2, Figure 3 and Figure 4 are listed in Table 1.

It can be seen from Table 1 that the initial heading can be obtained from Equation (19) when the accelerometer arrangements are given in Figure 3e and Figure 4a. $x_{1}$, $x_{2}$, and $x_{4}$ for the initial heading calculation for the accelerometer arrangement in Figure 3e are given in Equations (21)–(23), respectively.

(21) $$x_{1}=\frac{\sqrt{3}}{22r}\left(-3a_{1}-4a_{3}-3a_{4}+3a_{5}+4a_{6}-8a_{7}+3a_{8}\right)$$

(22) $$x_{2}=\frac{\sqrt{3}}{22r}\left(-4a_{1}+2a_{3}+7a_{4}+4a_{5}-2a_{6}+4a_{7}-7a_{8}\right)$$

(23) $$x_{4}=\frac{\sqrt{3}}{22r}\left(-5a_{1}-3a_{3}+6a_{4}-6a_{5}+3a_{6}+5a_{7}+5a_{8}\right)$$

where $a_{k}(k=1,\cdots ,8)$ is given in Equation (24). r denotes the distance from the center of gravity (accelerometer 9) to accelerometers 1 to 8.

(24) $$a_{k}=y_{k}-{\left[d_{k}^{b}\right]}^{T}g^{b}$$

$x_{1}$, $x_{2},$ and $x_{4}$ for the initial heading calculation for the accelerometer arrangement in Figure 4a are given in Equations (25)–(27), respectively.

(25) $$x_{1}=\frac{1}{2r}\left(a_{5}+a_{7}\right)$$

(26) $$x_{2}=\frac{1}{2r}\left(a_{6}+a_{10}\right)$$

(27) $$x_{4}=\frac{1}{2r}\left(a_{4}-a_{8}-a_{12}\right)$$

where $a_{k}(k=4,5,6,7,8,10,12)$ is given in Equation (24). r denotes the distance from the center of gravity (accelerometers 1 to 3) to accelerometers 4 to 12.

### 3.4. Initial Heading Error of Generic INS

The coarse alignment error characteristic of generic INS has been studied in many literature publications [1,3,5,21,22]. In this section, it is briefly introduced with the aim of deriving the initial heading error of the GF-INS.

The gyroscope output in the navigation frame can be expressed in Equation (28) when the vehicle is stationary.

(28) $${\tilde{\omega }}_{ie}^{n}=C_{b}^{n}\left(\omega_{ie}^{b}+\delta \omega_{ie}^{b}\right)=C_{b}^{n}\omega_{ie}^{b}+C_{b}^{n}\delta \omega_{ie}^{b}$$

where ${\tilde{\omega }}_{ie}^{n}$ denotes the gyroscope output in the navigation frame and $\delta \omega_{ie}^{b}$ the gyroscope output error in the body frame. If the roll, pitch, and heading are sufficiently small, $C_{b}^{n}$ can be expressed in Equation (29).

(29) $$C_{b}^{n}≅\left[\begin{array}{ccc} 1 & -\delta \psi & \delta \theta \\ \delta \psi & 1 & -\delta \phi \\ -\delta \theta & \delta \phi & 1 \end{array}\right]$$

where δϕ, δθ, and δψ denote sufficiently small roll, pitch, and heading, respectively. In order to make the description simple, it is assumed that the body frame and the navigation frame are the same, i.e., $\omega_{ie}^{b}=\omega_{ie}^{n}$. Then, Equation (28) can be written in Equation (30).

(30) $${\tilde{\omega }}_{ie}^{n}={\left[\begin{array}{ccc} {\left({\tilde{\omega }}_{ie}^{n}\right)}_{N} & {\left({\tilde{\omega }}_{ie}^{n}\right)}_{E} & {\left({\tilde{\omega }}_{ie}^{n}\right)}_{D} \end{array}\right]}^{T}\;\;\;\;\;\;\;\;\;\;\;\;\;≅\left[\begin{array}{c} \Omega \cos L-\delta \theta \cdot \Omega \sin L+{\left(\delta \omega_{ie}^{b}\right)}_{x}\\ \delta \psi \cdot \Omega \cos L+\delta \phi \cdot \Omega \sin L+{\left(\delta \omega_{ie}^{b}\right)}_{y}\\ -\delta \theta \cdot \Omega \cos L-\Omega \sin L+{\left(\delta \omega_{ie}^{b}\right)}_{z} \end{array}\right]$$

In order to obtain the initial heading, the east component of the gyroscope output (${\left({\tilde{\omega }}_{ie}^{n}\right)}_{E}$) should be zero, as in Equation (31).

(31) $${\left({\tilde{\omega }}_{ie}^{n}\right)}_{E}=\delta \psi \cdot \Omega \cos L+\delta \phi \cdot \Omega \sin L+{\left(\delta \omega_{ie}^{b}\right)}_{y}=0$$

δψ can be written in Equation (32) when δϕ=0 in Equation (31).

(32) $$\delta \psi =-\frac{{\left(\delta \omega_{ie}^{b}\right)}_{y}}{\Omega \cos L}$$

It can be seen from Equation (32) that the initial heading error in the generic INS is proportional to the gyroscope error of the east direction (the pitch axis direction) and inversely proportional to Ωcos⁡L when the body frame is the same as the navigation frame. Therefore, the heading error is minimum when the vehicle is located at the equator, and the heading error increases as the vehicle becomes nearer to the pole (L=±90°).

### 3.5. Initial Heading Error of GF-INS

Let us now consider the initial heading error of the GF-INS with the previous initial heading error of the general INS.

The accelerometer output ${\tilde{y}}_{k}$ of the GF-IMU can be represented in Equation (33).

(33) $${\tilde{y}}_{k}=y_{k}+\delta y_{k}$$

where $\delta y_{k}$ denotes the error of the k-th accelerometer output. If Equation (18) is used, $\hat{a}$ can be represented in Equation (34) from the accelerometer output ${\tilde{y}}_{k}$ in Equation (33) and ${\hat{g}}^{b}$ in Equation (11).

(34) $$\hat{a}=\left[\begin{array}{c} {\tilde{y}}_{1}+{\left[d_{1}^{b}\right]}^{T}{\hat{g}}^{b}\\ \vdots \\ {\tilde{y}}_{N}+{\left[d_{N}^{b}\right]}^{T}{\hat{g}}^{b} \end{array}\right]=H\hat{x}$$

where $\hat{x}$ is the estimate of x from the accelerometer outputs. Error δx of $\hat{x}$ can be represented in Equation (35) from Equations (18) and (34).

(35) $$\begin{array}{cc} \delta x & =\hat{x}-x={\left(H^{T}H\right)}^{-1}H^{T}\left(\hat{a}-a\right)\\ & ={\left(H^{T}H\right)}^{-1}H^{T}\left(\left[\begin{array}{c} {\tilde{y}}_{1}+{\left[d_{1}^{b}\right]}^{T}{\hat{g}}^{b}\\ \vdots \\ {\tilde{y}}_{N}+{\left[d_{N}^{b}\right]}^{T}{\hat{g}}^{b} \end{array}\right]-\left[\begin{array}{c} y_{1}+{\left[d_{1}^{b}\right]}^{T}g^{b}\\ \vdots \\ y_{N}+{\left[d_{N}^{b}\right]}^{T}g^{b} \end{array}\right]\;\right)\\ & ={\left(H^{T}H\right)}^{-1}H^{T}\left[\begin{array}{c} \delta y_{1}+{\left[d_{1}^{b}\right]}^{T}\delta g^{b}\\ \vdots \\ \delta y_{N}+{\left[d_{N}^{b}\right]}^{T}\delta g^{b} \end{array}\right]\\ & ={\left(H^{T}H\right)}^{-1}H^{T}{\left[\begin{array}{ccc} \delta a_{1} & \cdots & \delta a_{N} \end{array}\right]}^{T}\\ & ={\left(H^{T}H\right)}^{-1}H^{T}\delta a \end{array}$$

where δa and $\delta g^{b}$ denote the errors of $\hat{a}$ and ${\hat{g}}^{b}$, respectively. The covariance of δx**,** $\Sigma_{\delta x}$ can be obtained in Equation (36) from Equation (35).

(36) $$\Sigma_{\delta x}={\left(H^{T}\Sigma_{\delta a}^{-1}H\right)}^{-1}$$

where the covariance of $\delta a,\Sigma_{\delta a}$ is given in Equation (37) since $\mathrm{cov}\left[\delta a_{i},\delta a_{j}\right]=0$ if i≠j.

(37) $$\Sigma_{\delta a}=\left[\begin{array}{ccc} \sigma_{\delta a_{1}}^{2} & 0 & 0\\ 0 & \ddots & 0\\ 0 & 0 & \sigma_{\delta a_{N}}^{2} \end{array}\right]$$

where $\sigma_{\delta a_{k}}^{2}$ denotes the variance of $\delta a_{k}$. Let us calculate the variances of $\delta x_{1}$, $\delta x_{2}$, and $\delta x_{4}$ given in Equations (21)–(27). If accelerometers with the same specification are used, Equation (36) can be represented in Equation (38) since the condition $\sigma_{\delta a_{1}}^{2}=\cdots =\sigma_{\delta a_{N}}^{2}=\sigma_{\delta a}^{2}$ is valid.

(38) $$\Sigma_{\delta x}=\sigma_{\delta a}^{2}{\left(H^{T}H\right)}^{-1}$$

The variances of $\delta x_{1}$, $\delta x_{2}$, and $\delta x_{4}$ are listed in Table 2 for Figure 3e and Figure 4a from Equation (37).

The errors of $\hat{x_{1}}$, $\hat{x_{2}}$, and $\hat{x_{4}}$ in Equation (35) are given in Equation (39) when the body frame is the same as the navigation frame.

(39) $$\begin{array}{cc} \left[\begin{array}{c} \delta x_{1}\\ \delta x_{2}\\ \delta x_{4} \end{array}\right] & =\left[\begin{array}{c} \hat{x_{1}}\\ \hat{x_{2}}\\ \hat{x_{4}} \end{array}\right]-\left[\begin{array}{c} x_{1}\\ x_{2}\\ x_{4} \end{array}\right]=\left[\begin{array}{c} {\left({\tilde{\omega }}_{ie}^{b}\right)}_{x}{\left({\tilde{\omega }}_{ie}^{b}\right)}_{y}\\ {\left({\tilde{\omega }}_{ie}^{b}\right)}_{x}{\left({\tilde{\omega }}_{ie}^{b}\right)}_{z}\\ {\left({\tilde{\omega }}_{ie}^{b}\right)}_{x}^{2} \end{array}\right]-\left[\begin{array}{c} {\left(\omega_{ie}^{b}\right)}_{x}{\left(\omega_{ie}^{b}\right)}_{y}\\ {\left(\omega_{ie}^{b}\right)}_{x}{\left(\omega_{ie}^{b}\right)}_{z}\\ {\left(\omega_{ie}^{b}\right)}_{x}^{2} \end{array}\right]\\ & =(\Omega \cos L+{\left(\delta \omega_{ie}^{b}\right)}_{x})\left[\begin{array}{c} {\left(\delta \omega_{ie}^{b}\right)}_{y}\\ -\Omega \sin L+{\left(\delta \omega_{ie}^{b}\right)}_{z}\\ \Omega \cos L+{\left(\delta \omega_{ie}^{b}\right)}_{x} \end{array}\right]-\left[\begin{array}{c} 0\\ -\Omega^{2}\cos L\cdot \sin L\\ {\left(\Omega \cos L\right)}^{2} \end{array}\right]\\ & ≅\left[\begin{array}{c} \Omega \cos L{\left(\delta \omega_{ie}^{b}\right)}_{y}\\ -\Omega \sin L{\left(\delta \omega_{ie}^{b}\right)}_{x}+\Omega \cos L{\left(\delta \omega_{ie}^{b}\right)}_{z}\\ 2\Omega \cos L{\left(\delta \omega_{ie}^{b}\right)}_{x} \end{array}\right] \end{array}$$

Equation (32) can be represented as Equation (40) using Equation (39).

(40) $$\delta \psi =-\frac{{\left(\delta \omega_{ie}^{b}\right)}_{y}}{\Omega \cos L}=-\frac{{\Omega \cos L\left(\delta \omega_{ie}^{b}\right)}_{y}}{{\left(\Omega \cos L\right)}^{2}}=-\frac{\delta x_{1}}{{\left(\Omega \cos L\right)}^{2}}$$

It can be seen from Equation (39) that the heading error is proportional to $\delta x_{1},$ which is the error of ${\left(\omega_{ie}^{b}\right)}_{x}{\left(\omega_{ie}^{b}\right)}_{y}$ obtained from Equation (19), and it is impossible to get the heading at the pole as with Equation (32). As shown in Table 2, $\delta x_{1}$ depends on the grade and arrangement of the accelerometers. 

Let us examine the heading error quantitatively by inserting the error specification of actual accelerometers into Equation (40). Table 3 shows the error specification of QA3000, a representative navigation-grade accelerometer manufactured by Honeywell Inc., USA [23] and Absolute Quantum Gravimeter, which is known as the most accurate accelerometer manufactured by Muquans Inc. (Talence, France) [24].

Let us obtain the initial heading error for the accelerometers in Table 3 when L=0° and r=1 m. In order to check the effect of the accelerometer error on the heading error, δx can be expressed in the accelerometer error δy as Equation (41) when ${\hat{g}}^{b}=g^{b}$ in Equation (30).

(41) $$\delta x={\left(H^{T}H\right)}^{-1}H^{T}{\left[\begin{array}{ccc} \delta y_{1} & \cdots & \delta y_{N} \end{array}\right]}^{T}={\left(H^{T}H\right)}^{-1}H^{T}\delta y$$

Since $\sigma_{\delta a}^{2}=\sigma_{\delta y}^{2}{=\sigma }_{{\delta y}_{i}}^{2}(i=1,\cdots ,N$), 1σ of $\delta x_{1}$ can be obtained when the specifications of the accelerometers in Table 3 are inserted into the expression in Table 2. By inserting these values into Equation (40), the 1 σ values of the initial heading errors are given in Table 4.

It can be seen from Table 4 that the practical initial heading of GF-INS cannot be obtained, even when the absolute quantum gravimeter, which is the most accurate accelerometer at the present technology level, and/or the navigation-grade accelerometer are used since the heading error is very large. For the 1 σ of the heading error in the arrangement of Figure 4a to be less than 5°, the accuracy of the accelerometer has to be less than $\sigma_{\delta y}=6.696\times 10^{-11}$ G. This value is 15 times more accurate than the absolute quantum gravimeter in Table 3. It can be seen from these results that it is difficult to have an initial heading with the required performance in the GF-INS, and these results are similar to those mentioned by Vaknin and Klein, in which the initial heading cannot be obtained [7,9].

## 4. Coarse Alignment of GF-INS with Gyroscope

According to the results in Section 3, even when the GF-IMU is configured with extremely accurate accelerometers, it is impossible to obtain an initial heading in the GF-INS. In this section, the heading error is examined when gyros are added to the GF-INS.

The gyroscope output ${\tilde{g}}_{n}$ can be expressed in Equation (42) in the body frame.

(42) $${\tilde{g}}_{n}=g_{n}+\delta g_{n}$$

where $\delta g_{n},(n=x,y,orz)$ denotes the n-axis gyroscope output error in the body frame.

The relationship between the gyroscope output and x in Equation (18) is given in Equation (43).

(43) $$b={\left[\begin{array}{cccccc} b_{1} & b_{2} & b_{3} & b_{4} & b_{5} & b_{6} \end{array}\right]}^{T}={\left[\begin{array}{cccccc} g_{x}g_{y} & g_{x}g_{z} & g_{y}g_{z} & g_{x}^{2} & g_{y}^{2} & g_{z}^{2} \end{array}\right]}^{T}=I_{6\times 6}x$$

where $I_{6\times 6}$ denotes a 6×6 unit matrix. Inserting Equation (42) into Equation (43), the error of $\tilde{b}$ is given in Equation (44).

(44) $$\delta b=\tilde{b}-b=\left[\begin{array}{c} \delta b_{1}\\ \delta b_{2}\\ \delta b_{3}\\ \delta b_{4}\\ \delta b_{5}\\ \delta b_{6} \end{array}\right]=\left[\begin{array}{c} {\tilde{g}}_{x}{\tilde{g}}_{y}\\ {\tilde{g}}_{x}{\tilde{g}}_{z}\\ {\tilde{g}}_{y}{\tilde{g}}_{z}\\ {\tilde{g}}_{x}^{2}\\ {\tilde{g}}_{y}^{2}\\ {\tilde{g}}_{z}^{2} \end{array}\right]-\left[\begin{array}{c} g_{x}g_{y}\\ g_{x}g_{z}\\ g_{y}g_{z}\\ g_{x}^{2}\\ g_{y}^{2}\\ g_{z}^{2} \end{array}\right]=\left[\begin{array}{c} g_{x}\delta g_{y}+g_{y}\delta g_{x}+\delta g_{x}\delta g_{y}\\ g_{x}\delta g_{z}+g_{z}\delta g_{x}+\delta g_{x}\delta g_{z}\\ g_{y}\delta g_{z}+g_{z}\delta g_{y}+\delta g_{y}\delta g_{z}\\ 2g_{x}\delta g_{x}+\delta g_{x}^{2}\\ 2g_{y}\delta g_{y}+\delta g_{y}^{2}\\ 2g_{z}\delta g_{z}+\delta g_{z}^{2} \end{array}\right]$$

It can be observed from Equation (44) that the error of $\tilde{b}$ is proportional to the gyroscope outputs $g_{x}$, $g_{y}$, and $g_{z}$.

For the simplicity of the description, consider the case that L=0° and the body frame is the same as the navigation frame. In this case, Equation (44) can be represented in Equation (45) since ${\left[\begin{array}{ccc} g_{x} & g_{y} & g_{z} \end{array}\right]}^{T}={\left[\begin{array}{ccc} \Omega & 0 & 0 \end{array}\right]}^{T}$.

(45) $$\delta b=\tilde{b}-b=\left[\begin{array}{c} \delta b_{1}\\ \delta b_{2}\\ \delta b_{3}\\ \delta b_{4}\\ \delta b_{5}\\ \delta b_{6} \end{array}\right]=\left[\begin{array}{c} \Omega \delta g_{y}+\delta g_{x}\delta g_{y}\\ \Omega \delta g_{z}+\delta g_{x}\delta g_{z}\\ \delta g_{y}\delta g_{z}\\ 2\Omega \delta g_{x}+\delta g_{x}^{2}\\ \delta g_{y}^{2}\\ \delta g_{z}^{2} \end{array}\right]≅\left[\begin{array}{c} \Omega \delta g_{y}\\ \Omega \delta g_{z}\\ 0\\ 2\Omega \delta g_{x}\\ 0\\ 0 \end{array}\right]$$

x can be estimated in Equation (46) using $\hat{a}$ calculated from the accelerometer outputs and gyroscope outputs $\tilde{b}$.

(46) $$\hat{x}={\left(H_{aug}^{T}WH_{aug}\right)}^{-1}{H_{aug}}^{T}W\left[\begin{array}{c} \hat{a}\\ \tilde{b} \end{array}\right]={\left(H_{aug}^{T}WH_{aug}\right)}^{-1}{H_{aug}}^{T}Wc$$

where W denotes the weighting matrix given in Equation (47).

(47) $$W={\left(\Sigma_{\delta c}\right)}^{-1}={\mathrm{diag}\left(\underset{N}{\underbrace{\sigma_{\delta a}^{2},\cdots ,\sigma_{\delta a}^{2}}},\sigma_{\delta b_{1}}^{2},\cdots ,\sigma_{\delta b_{6}}^{2}\right)}^{-1}$$

where $\Sigma_{\delta c}$ is the covariance of δc, and $\sigma_{\delta b_{k}}^{2}$ is the variance of $\delta b_{k}$. The measurement matrix $H_{aug}$ is given in Equation (48).

(48) $$H_{aug}=\left[\begin{array}{c} H_{a}\\ H_{b} \end{array}\right]=\left[\begin{array}{c} \begin{array}{ccc} {\left(h_{1}\right)}_{1} & \cdots & {\left(h_{1}\right)}_{6}\\ \vdots & \ddots & \vdots \\ {\left(h_{N}\right)}_{1} & \cdots & {\left(h_{N}\right)}_{6} \end{array}\\ I_{6\times 6} \end{array}\right]$$

where $H_{a}$ and $H_{b}$ are the measurement matrices of the accelerometer output and the gyroscope output, respectively.

The number of arrangements of gyroscopes, in which the gyroscopes are located at one or two axes among the roll, pitch, and yaw axes, is six in the body frame. One gyroscope can be located at the roll, pitch, or yaw axis, and two gyroscopes can be located at the roll and pitch axes, roll and yaw axes, or pitch and yaw axes. $\tilde{b}$, W, and $H_{b}$ in Equation (46) for the gyroscope arrangements are listed in Table 5. The error covariance of the estimated x in Equation (46) is given in Equation (49).

(49) $$\Sigma_{\delta x}={\left(H_{aug}^{T}WH_{aug}\right)}^{-1}$$

For the accelerometer arrangement in Figure 4a, the heading error can be checked when the initial heading is obtained using the accelerometer outputs and the added gyroscope outputs. The variances of $\delta x_{1}$, $\delta x_{2}$, and $\delta x_{4}$ from Equation (49) are listed in Table 6.

It can be seen from Table 6 that the variance of $\delta x_{1}$ is the same as the case when only accelerometers are used, except for the case when the added gyroscopes are located at the roll and pitch axes. Since the heading error is determined by $\delta x_{1}$ as shown in Equation (39), it can be seen that the initial heading accuracy is determined by the performance of the gyros located at the roll and pitch axes when the gyroscopes are added to the GF-INS.

Let us obtain the variance of $\delta x_{1}$ to the grade of the gyroscope in order to have the initial heading error. First, the variance of $\delta b_{1}$ in Equation (45) is given in Equation (50).

(50) $$\begin{array}{cc} \sigma_{\delta b_{1}}^{2} & =\mathrm{var}\left[\delta b_{1}\right]=\mathrm{var}\left[\Omega \delta g_{y}\right]\\ & =\Omega^{2}\mathrm{var}\left[\delta g_{y}\right]\\ & =\Omega^{2}\sigma_{\delta g_{y}}^{2} \end{array}$$

When the gyroscopes with the same error specifications are located at the roll and pitch axes, the variance of $\delta x_{1}$ is in Equation (51) since $\sigma_{\delta g_{x}}^{2}=\sigma_{\delta g_{y}}^{2}=\sigma_{\delta g}^{2}$.

(51) $$\sigma_{\delta x_{1}}^{2}=\frac{\sigma_{\delta a}^{2}\sigma_{\delta b_{1}}^{2}}{\sigma_{\delta a}^{2}+2r^{2}\sigma_{\delta b_{1}}^{2}}=\frac{\Omega^{2}\sigma_{\delta a}^{2}\sigma_{\delta g}^{2}}{\sigma_{\delta a}^{2}+2r^{2}\Omega^{2}\sigma_{\delta g}^{2}}$$

As shown in Equation (50), the variance of $\delta x_{1}$ depends on the gyroscope performance ($\sigma_{\delta g}^{2}$). Table 7 shows the bias error of Honeywell’s tactical-grade IMU, HG1700AG58, and navigation-grade gyro, GG1320AN [25,26]. It is known that the initial heading cannot be obtained when sub-tactical-grade gyroscopes that cannot accurately measure the Earth rate are used. For this reason, the specifications of Honeywell’s tactical- and navigation-grade gyros, which are widely used in guided weapons and navigation systems, are used in the error calculation.

Table 8 shows the 1 σ values of the heading error calculated using Equation (51) for the accelerometer error specifications from Table 3 and the gyroscope error specifications in Table 7 into Equation (51) when L=0° and r=1 m, as in Section 3.4. This is the same result as that of the generic INS gyro-compassing [1,4]. It can be seen from this result in Table 8 that the initial heading error is more dependent on the performance of the gyro than the performance of the accelerometer. This suggests that the practical initial heading cannot be obtained by using only GF-IMU without additional sensors, such as a gyroscope.

## 5. Concluding Remarks and Further Studies

In this note, the feasibility of obtaining the initial attitude of the GF-INS has been investigated by deriving the equation for the initial attitude from the accelerometer outputs. The initial heading is expressed in the accelerometer outputs of two configurations, which satisfies a specific condition among 15 GF-IMU configurations presented in the literature. In particular, it has been clarified that it is essential in the initial heading alignment as the general INS to accurately measure the Earth rate by analytically deriving the initial heading error due to the arrangement and error of the accelerometers. The initial heading errors have been checked for two kinds of GF-IMUs, which are composed of representative navigation-grade accelerometers and absolute quantum gravimeters, which are known as the most accurate accelerometers. The errors have been calculated by inserting error specifications into initial heading error equations. The results show that the initial heading cannot be obtained within a practical error level by using only GF-IMU due to limitations of the accelerometer production technology. In addition to this, the initial heading error has been checked when gyroscopes are used with the GF-IMU. It has been checked from this result that the initial heading accuracy depends more on the performance of the gyroscope than that of the accelerometer

It can be expected from the results that it is difficult to determine the initial heading using only the GF-IMU, even though the manufacturing technology of the accelerometer has improved. If the initial heading is needed in the GF-INS, non-inertial adding sensors, such as a magnetic compass or the attitude-determination GNSS receiver with two antennas, can be combined with the GF-IMU.

## Figures and Tables

**Table 1:** Rank of $H^{T}H$.

Accelerometer Configuration	Number of Accelerometers	Rank ⁡HTH
Figure 1a [9,10,12]	6	3
Figure 1b [9]	6	3
Figure 1c [9]	6	5
Figure 1d [11]	6	2
Figure 1e [11]	6	3
Figure 1f [11]	6	3
Figure 2 [16]	7	4
Figure 3a [12]	9	3
Figure 3b [9]	9	3
Figure 3c [13,14]	9	3
Figure 3d [13]	9	3
Figure 3e [9]	9	6
Figure 3f [9]	9	5
Figure 4a [9,15]	12	6
Figure 4b [17]	12	3

**Table 2:** Variances of $\delta x_{1}$, $\delta x_{2}$ and $\delta x_{4}$.

	Configuration	Figure 3e	Figure 4a
Variance of $\delta x_{k}$			
$\sigma_{\delta x_{1}}^{2}$		$\frac{9}{11r^{2}}\sigma_{\delta a}^{2}$	$\frac{1}{2r^{2}}\sigma_{\delta a}^{2}$
$\sigma_{\delta x_{2}}^{2}$		$\frac{21}{22r^{2}}\sigma_{\delta a}^{2}$	$\frac{1}{2r^{2}}\sigma_{\delta a}^{2}$
$\sigma_{\delta x_{4}}^{2}$		$\frac{45}{44r^{2}}\sigma_{\delta a}^{2}$	$\frac{3}{4r^{2}}\sigma_{\delta a}^{2}$

**Table 3:** Specification of accelerometers.

Model Number	Manufacturer	Error Spec. (1 σ Bias)	Type
QA3000	Honeywell Inc., USA	$25\times 10^{-6}$ G (G = 9.8 m/s2)	Quartz Pendulum
Absolute Quantum Gravimeter	Muquans, France	$10^{-9}$ G	Laser-Cooled Atom

**Table 4:** The 1σ value of initial heading error.

	Configuration	Figure 3e	Figure 4a
Model Number			
QA3000		$2.39\times 10^{6}$°	$1.87\times 10^{6}$°
Absolute Quantum Gravimeter		95.51°	74.67°

**Table 5:** Value of $\tilde{b}$ **W** and **H_b_** for gyro configurations.

Gyro Axis	$\tilde{b}$	W	$H_{b}$
Roll	$b_{4}$	${\mathrm{diag}\left(\sigma_{\delta a_{1}}^{2},\cdots ,\sigma_{\delta a_{N}}^{2},\sigma_{\delta b_{4}}^{2}\right)}^{-1}$	$\left[\begin{array}{cccccc} 0 & 0 & 0 & 1 & 0 & 0 \end{array}\right]$
Pitch	$b_{5}$	${\mathrm{diag}\left(\sigma_{\delta a_{1}}^{2},\cdots ,\sigma_{\delta a_{N}}^{2},\sigma_{\delta b_{5}}^{2}\right)}^{-1}$	$\left[\begin{array}{cccccc} 0 & 0 & 0 & 0 & 1 & 0 \end{array}\right]$
Yaw	$b_{6}$	${\mathrm{diag}\left(\sigma_{\delta a_{1}}^{2},\cdots ,\sigma_{\delta a_{N}}^{2},\sigma_{\delta b_{6}}^{2}\right)}^{-1}$	$\left[\begin{array}{cccccc} 0 & 0 & 0 & 0 & 0 & 1 \end{array}\right]$
Roll and Pitch	${\left[\begin{array}{ccc} b_{1} & b_{4} & b_{5} \end{array}\right]}^{T}$	${\mathrm{diag}\left(\sigma_{\delta a_{1}}^{2},\cdots ,\sigma_{\delta a_{N}}^{2},\sigma_{\delta b_{1}}^{2},\sigma_{\delta b_{4}}^{2},\sigma_{\delta b_{5}}^{2}\right)}^{-1}$	$\left[\begin{array}{cccccc} 1 & 0 & 0 & 0 & 0 & 0\\ 0 & 0 & 0 & 1 & 0 & 0\\ 0 & 0 & 0 & 0 & 1 & 0 \end{array}\right]$
Roll and Yaw	${\left[\begin{array}{ccc} b_{2} & b_{4} & b_{6} \end{array}\right]}^{T}$	${\mathrm{diag}\left(\sigma_{\delta a_{1}}^{2},\cdots ,\sigma_{\delta a_{N}}^{2},\sigma_{\delta b_{2}}^{2},\sigma_{\delta b_{4}}^{2},\sigma_{\delta b_{6}}^{2}\right)}^{-1}$	$\left[\begin{array}{cccccc} 0 & 1 & 0 & 0 & 0 & 0\\ 0 & 0 & 0 & 1 & 0 & 0\\ 0 & 0 & 0 & 0 & 0 & 1 \end{array}\right]$
Pitch and Yaw	${\left[\begin{array}{ccc} b_{3} & b_{5} & b_{6} \end{array}\right]}^{T}$	${\mathrm{diag}\left(\sigma_{\delta a_{1}}^{2},\cdots ,\sigma_{\delta a_{N}}^{2},\sigma_{\delta b_{3}}^{2},\sigma_{\delta b_{5}}^{2},\sigma_{\delta b_{6}}^{2}\right)}^{-1}$	$\left[\begin{array}{cccccc} 0 & 0 & 1 & 0 & 0 & 0\\ 0 & 0 & 0 & 0 & 1 & 0\\ 0 & 0 & 0 & 0 & 0 & 1 \end{array}\right]$

**Table 6:** Variances of $\delta x_{1}$, $\delta x_{2}$ and $\delta x_{4}$.

	$\mathrm{Variance}\;\mathrm{of}\;{\delta x}_{k}$	$\sigma_{\delta x_{1}}^{2}$	$\sigma_{\delta x_{2}}^{2}$	$\sigma_{\delta x_{4}}^{2}$
Gyro Axis				
Roll		$\frac{1}{2r^{2}}\sigma_{\delta a}^{2}$	$\frac{1}{2r^{2}}\sigma_{\delta a}^{2}$	$\frac{3\sigma_{\delta a}^{2}\sigma_{\delta b_{4}}^{2}}{{3\sigma }_{\delta a}^{2}+4r^{2}\sigma_{\delta b_{4}}^{2}}$
Pitch		$\frac{1}{2r^{2}}\sigma_{\delta a}^{2}$	$\frac{1}{2r^{2}}\sigma_{\delta a}^{2}$	$\frac{\sigma_{\delta a}^{2}\left(2\sigma_{\delta a}^{2}+3r^{2}\sigma_{\delta b_{5}}^{2}\right)}{r^{2}\left({3\sigma }_{\delta a}^{2}+4r^{2}\sigma_{\delta b_{5}}^{2}\right)}$
Yaw		$\frac{1}{2r^{2}}\sigma_{\delta a}^{2}$	$\frac{1}{2r^{2}}\sigma_{\delta a}^{2}$	$\frac{\sigma_{\delta a}^{2}\left(2\sigma_{\delta a}^{2}+3r^{2}\sigma_{\delta b_{6}}^{2}\right)}{r^{2}\left({3\sigma }_{\delta a}^{2}+4r^{2}\sigma_{\delta b_{6}}^{2}\right)}$
Roll and Pitch		$\frac{\sigma_{\delta a}^{2}\sigma_{\delta b_{1}}^{2}}{\sigma_{\delta a}^{2}+2r^{2}\sigma_{\delta b_{1}}^{2}}$	$\frac{1}{2r^{2}}\sigma_{\delta a}^{2}$	$\frac{\sigma_{\delta a}^{2}\sigma_{\delta b_{4}}^{2}\left(2\sigma_{\delta a}^{2}+r^{2}3\sigma_{\delta b_{5}}^{2}\right)}{{3\sigma }_{\delta a}^{2}+r^{2}\left({3\sigma }_{\delta a}^{2}\sigma_{\delta b_{4}}^{2}+{3\sigma }_{\delta a}^{2}\sigma_{\delta b_{5}}^{2}+4r^{2}\sigma_{\delta b_{4}}^{2}\sigma_{\delta b_{5}}^{2}\right)}$
Roll and Yaw		$\frac{1}{2r^{2}}\sigma_{\delta a}^{2}$	$\frac{\sigma_{\delta a}^{2}\sigma_{\delta b_{2}}^{2}}{\sigma_{\delta a}^{2}+2r^{2}\sigma_{\delta b_{2}}^{2}}$	$\frac{\sigma_{\delta a}^{2}\sigma_{\delta b_{4}}^{2}\left(2\sigma_{\delta a}^{2}+3r^{2}\sigma_{\delta b_{6}}^{2}\right)}{{2\sigma }_{\delta a}^{2}+r^{2}\left({3\sigma }_{\delta a}^{2}\sigma_{\delta b_{4}}^{2}+{3\sigma }_{\delta a}^{2}\sigma_{\delta b_{6}}^{2}+4r^{2}\sigma_{\delta b_{4}}^{2}\sigma_{\delta b_{6}}^{2}\right)}$
Pitch and Yaw		$\frac{1}{2r^{2}}\sigma_{\delta a}^{2}$	$\frac{1}{2r^{2}}\sigma_{\delta a}^{2}$	$\frac{\sigma_{\delta a}^{2}\left({\left(\sigma_{\delta a}^{2}\right)}^{2}+2r^{2}\sigma_{\delta a}^{2}\sigma_{\delta b_{5}}^{2}+2r^{2}\sigma_{\delta a}^{2}\sigma_{\delta b_{6}}^{2}+3r^{4}\sigma_{\delta b_{5}}^{2}\sigma_{\delta b_{6}}^{2}\right)}{r^{2}\left({{2\left(\sigma_{\delta a}^{2}\right)}^{2}+3r^{2}\sigma }_{\delta a}^{2}\sigma_{\delta b_{5}}^{2}+{3r^{2}\sigma }_{\delta a}^{2}\sigma_{\delta b_{6}}^{2}+4r^{4}\sigma_{\delta b_{5}}^{2}\sigma_{\delta b_{6}}^{2}\right)}$

**Table 7:** Specification of gyroscope.

Gyro	Error Spec. (1 σ Bias)	Type
RLG in HG1700AG58 (Honeywell Inc., USA)	1°/h	Tactical-Grade RLG
GG1320AN (Honeywell Inc., USA)	0.003°/h	Navigation-Grade RLG

**Table 8:** The 1 σ value of heading error.

	Gyro	RLG in HG1700AG58	GG1320AN
Accelerometer			
QA3000		3.809°	0.011°
Absolute Quantum Gravimeter		3.804°	0.011°

## Data Availability

Not applicable.
